# Editorial: Promotion of quality of life in oncology patients and survivors through physical activity

**DOI:** 10.3389/fonc.2025.1581264

**Published:** 2025-03-17

**Authors:** Sarah J. Hardcastle, Marta Leyton Roman, Ruth Jiménez-Castuera

**Affiliations:** ^1^ School of Sport and Physical Activity, Sheffield Hallam University, Sheffield, United Kingdom; ^2^ Institute for Health Research, The University of Notre Dame Australia, Fremantle, WA, Australia; ^3^ Department of Didactics of Musical, Plastic and Body Expression, University of Extremadura, Caceres, Spain

**Keywords:** exercise oncology, cancer, intervention, survivorship, exercise prescription, health promotion

Exercise oncology is a rapidly expanding field with a wealth of research evidencing the benefits of physical activity (PA) for health-related quality of life (HRQoL) and increasing evidence of improved survival for physically active cancer survivors postdiagnosis (1–3). The articles in this Research Topic provide important considerations in exercise oncology including (i) consideration of the distinct timepoints for exercise intervention and the role of exercise as a monotherapy, concurrent, maintenance or salvage therapy; (ii) consideration of the broad role of exercise at particular timepoints; (iii) the importance of efforts to engage underserved and disadvantaged cancer survivors who have the most to gain from exercise engagement; and (iv) the importance of challenging and changing misperceptions concerning the safety of exercise for cancer patients and their capability to exercise.

Courneya et al. propose a new framework: *Exercise Across the Postdiagnosis Cancer Continuum* (EPiCC) to encourage a more refined approach to the study of exercise from diagnosis onwards including distinct timepoints for intervention and the specific role(s) of exercise. Courneya et al. distinguish between two broad roles of exercise in cancer care: supportive care and disease treatment. The former is focused on HRQoL issues such as physical function, treatment tolerance, treatment side-effect management and psychosocial outcomes whereas the latter is centered on outcomes such as cancer control, disease control and survival. The EPiCC framework features six distinct cancer treatment-related time periods for exercise: before treatments, during treatments, between treatments, immediately after successful treatment(s), during the survivorship phase (following successful treatment), and during end-of-life care after unsuccessful treatments. Courneya et al. contend that the effects of exercise on the various outcomes will vary depending upon its positioning within differing cancer treatment combinations and that the model provides a structure to consider the potential roles of exercise. For example, prior to nonsurgical therapy, exercise could serve as a ‘priming monotherapy’ to support the effectiveness of subsequent treatments. Exercise could also be used as concurrent therapy alongside treatments or as ‘bridging monotherapy’ to control cancer in between treatments. Following successful treatment, exercise may serve as a ‘maintenance monotherapy’ for disease control and improved survival. The EPiCC framework is an important contribution to the field and provides a more sophisticated approach for future research within exercise oncology.

In addition to considerations of the role(s) of exercise and the distinct timepoint for intervention across the cancer continuum, future research would be worthwhile that ensures that participants are recruited for intervention based on the need for change (i.e., insufficiently physically active), and, on the premise that there is clear room for improvement in the primary outcome. Indeed, findings from the PPARCS trial by Hardcastle et al. found no improvements in HRQoL, likely due to survivors reporting a relatively high HRQoL at baseline leaving little room for improvement.

For improved impact, it is also important to increase our efforts to promote PA to underserved and socially disadvantaged cancer patients. The THRIVE protocol in this Research Topic by Yan et al. is an example of one such novel study that will examine the effectiveness of an exercise intervention on PA and cardiovascular disease risk among Hispanic/Latinx and Black cancer patients receiving chemotherapy for breast, prostate or colorectal cancer. As noted by the authors, ethnic minority groups tend to be underrepresented in clinical trials and may benefit from home-based exercise interventions to overcome some common barriers to exercise engagement including cost and access to exercise facilities (4–6) that are magnified for disadvantaged populations. The trial will compare the effects of a virtually supervised exercise intervention (3 x per week via Zoom) involving high-intensity interval training (HIIT) and resistance training to an unsupervised HIIT exercise intervention (with weekly calls), and an attention control condition. Such remotely delivered PA interventions have demonstrated promise to increase PA amongst cancer survivors (7–9).

Borsati et al. examined population perceptions of exercise oncology in Italy (n= 838). The authors found that despite positive attitudes concerning the value of exercise during treatment, only 40.2% agreed that exercise was safe and 27.2% believed that patients were capable of exercising during treatment. Further, only 9% believed that it was easy for patients to exercise during treatment. As the authors note, it is concerning that most participants were unsure of the safety of exercise for cancer patients and efforts are needed to ensure that policy makers, clinicians, patients and caregivers understand the high safety profile of PA for cancer (10, 11) and the importance of exercise during cancer treatment to improve cancer-related fatigue (12), physical function/QoL (13), disease management (e.g., through improved immune surveillance, reduced inflammation, improved insulin sensitivity) (14) and in the longer-term, improved survival (1–3). The perceptions of low capability for exercise may be related to misperceptions about what constitutes exercise (i.e., that exercise must be of vigorous intensity), low motivation, and/or a lack of knowledge on the benefits of PA for cancer patients. Cancer survivors lacking in confidence and motivation for exercise also report a preference for lower-intensity exercise (15). Future interventions that assess exercise intensity preferences and endeavor to match PA appropriately would be worthwhile to increase self-efficacy and PA engagement.

The collection of articles in this Research Topic highlights the diversity of research and perspectives within exercise oncology. The novel EPICC framework by Courneya et al. paves the way for a more sophisticated approach to examining the various role(s) of exercise at distinct timepoints along the cancer continuum, whilst Yan et al. and Hardcastle et al. focus on the effectiveness of novel distance-based intervention to increase PA and improve HRQoL in underserved and disadvantaged cancer survivors. Finally, research by Borsati et al. indicates that concerns of safety and capability may be key barriers to exercise engagement. Clearly, there is still much work needed to advocate the safety, tolerability and importance of exercise for cancer patients, such that clinicians, healthcare professionals and all those working in cancer care will be willing to promote PA to cancer patients, as an integral part of cancer treatment, and encourage cancer survivors to be physically active from the point of diagnosis onwards (16).
